# mHealth Apps in the Digital Marketplace for Pediatric Patients With Cancer: Systematic Search and Analysis

**DOI:** 10.2196/58101

**Published:** 2024-10-01

**Authors:** Micah A Skeens, Daniel I Jackson, Malcolm S Sutherland-Foggio, Emre Sezgin

**Affiliations:** 1 The Abigail Wexner Research Institute, Nationwide Children’s Hospital, 700 Children’s Dr, Columbus, OH, 43205, United States, 16147220000; 2 Department of Pediatrics, The Ohio State University College of Medicine, Columbus, OH, United States

**Keywords:** mHealth, mobile health, mobile application, mobile apps, digital health, digital technology, digital intervention, smartphones, cancer, oncology, pediatric cancer, paediatric cancer, pediatric oncology, paediatric oncology, systematic analysis, systematic analyses, review, mobile phone

## Abstract

**Background:**

The substantial increase in smartphone ownership has led to a rise in mobile health (mHealth) app use. Developing tailored features through mHealth apps creates a pathway to address the health care needs of pediatric patients with cancer and their families who have complex care needs. However, few apps are designed specifically to integrate with pediatric cancer care.

**Objective:**

This study reports a systematic search and analysis of mHealth apps available on the Apple App (iOS) and Google Play (Android) stores designed for pediatric cancer through a list of features that serve (1) patients, (2) caregivers, or (3) both audiences.

**Methods:**

Following PRISMA (Preferred Reporting Items for Systematic Reviews and Meta-Analyses) guidelines, we reviewed apps for pediatric patients with cancer and caregivers available as of January 30, 2024. We searched the Apple App and Google Play stores with a list of keyword combinations focusing on pediatric cancer care. The inclusion criteria were (1) specifically apps targeted toward pediatric patients with cancer, their families, or both; (2) available in either app store; and (3) available in English. Apps were assessed using the Mobile Application Rating Scale (MARS). The MARS is a quality assessment for mHealth apps, including components of engagement, functionality, aesthetics, and informational quality (5-point Likert scale items—1: low and 5: high quality).

**Results:**

In total, 22 apps were identified and 17 of those apps were available on both platforms. The most popular features (n=12) were resource sharing, symptom tracking, reminders, care team connections, journaling, community support, medication tracking, data visualizations, and appointment tracking. Features and interfaces were designed for caregivers (n=9) more frequently than the patients (n=7) while a subset of apps created options for both users (n=6). A total of 16 apps received positive reviews (mean 4.4, SD 0.59; Min=3.1, Max=5.0). A small subset (n=3) achieved over 5000 downloads; however, the majority (n=15) had fewer than 500. More than half (n=12) of the apps were not available in English. Apps requested access to a range of device functionalities to operate (mean 2.72, SD 3.13; Min=0, Max=10). Out of 22, a total of 17 apps were publicly accessible. The mean MARS scores for the apps ranged from 1.71 (SD 0.75) to 4.33 (SD 0.82). Overall, apps scored high on functionality (mean 3.72, SD 0.54) but low on engagement (mean 3.02, SD 0.93).

**Conclusions:**

Our review highlights the promising yet underdeveloped potential of mHealth apps in pediatric oncology care, underscoring the need for more inclusive, comprehensive, and integrative digital health solutions. Future developments should actively involve key stakeholders from the pediatric oncology community, including patients, families, and health care professionals, to ensure the apps meet specific needs while addressing linguistic and cultural barriers.

## Introduction

Approximately 400,000 children are diagnosed with cancer each year internationally, and the incidence continues to rise annually [1]. Fortunately, treatment advances have also resulted in significant improvements in survival rates [2,3]. Despite these significant advances, pediatric cancer remains a leading cause of death among children [4]. The management of pediatric cancer is complex and requires multidisciplinary care that involves ongoing monitoring, management of physical or psychological symptoms, and social support for families. This includes community resources, symptom management, rehabilitation, and access to educational content for patients and their families. Digital interventions, such as mobile health (mHealth) apps, have the potential to meet these needs in real time while eliminating barriers like the distance from a medical center, lifestyle demands (eg, work and school), and mental health stigma [5].

mHealth apps are software apps designed to run on mobile devices, such as smartphones and tablets [6]. The significant increase in mobile or smartphone ownership has simultaneously led to a rise in the use of mHealth apps. Furthermore, the global proliferation of mobile devices among the younger demographic underscores the feasibility of mHealth apps for pediatric patients with cancer. Recent statistics indicate that technological access is substantial among the adolescent cohort [7]. Approximately 95% of adolescents possess or at least have access to smartphones and 90% have access to a desktop or laptop computer in the United States. Notably, almost half (46%) of this demographic reported to be online almost constantly [7]. mHealth apps offer a range of health-related services and resources, such as tracking symptoms, providing medication reminders, and connecting patients with health care professionals [5,8]. The use of mHealth apps in the context of pediatric cancer is an emerging field that holds great potential for improving the management and outcomes of this disease [5,9]. mHealth can provide valuable support for families dealing with pediatric cancer, including access to emergency contact information, educational resources, and social support networks [10]. Some apps also provide pediatric cancer families with tools to improve psychosocial well-being and health outcomes [11]. Additionally, these apps can help health care professionals to monitor and track patient progress more efficiently while providing more personalized care [12].

Despite the increasing number of mHealth apps developed for patients with cancer, there is a lack of literature on pediatric cancer regarding the mHealth apps available on the market. To our knowledge, a limited literature has focused on mHealth apps for caregivers of pediatric patients with cancer [5,9]. One of the early investigations highlighted cancer apps for adolescent and young adult patients with cancer with their functionalities for symptom tracking, pain management, monitoring, and medication management [13]. Looking at the broader literature, the studies mostly reported findings on a specific mHealth app, which might be focusing on electronic medical diaries for mood, symptom and treatment tracking [14], care after cancer treatment [15], pre-rehabilitation support [16], and posttreatment medical adherence [17]. In addition, newer mHealth technologies have leveraged wearable technologies for tracking physical activity [18,19], social media behaviors [19,20], web-text messaging for weight management [21], and gamification of monitoring symptoms to address cancer-associated pain through self-guidance [22,23].

mHealth apps for pediatric patients require further investigation to explore the potential benefits collectively. Therefore, a broader perspective (beyond the apps available in the current literature) is required to understand the current state of the mHealth apps. In line with that, the evaluation of those apps further contributes to the current state of the app market in pediatric oncology care. The aim of this systematic search is (1) to investigate currently available mHealth apps designed specifically for pediatric oncology in mobile app repositories (Google Play store and Apple App store); (2) to analyze the features and cost of services provided; and (3) to conduct a descriptive analysis to inform developers, designers, and clinician scientists. Our study aims to evaluate pediatric cancer–specific mHealth apps that ultimately improve psychosocial and health outcomes in vulnerable populations.

## Methods

### Overview

We performed an observational, cross-sectional, descriptive study of all smartphone apps associated with pediatric cancer available on the iOS (Apple App store) and Android (Google Play store) platforms. We only evaluated apps available in these 2 online stores.

### Ethical Considerations

Institutional review board approval was not obtained as this is not human subjects research per our institutional policy and does not require institutional review board approval.

### Mobile App Search

The methodology used was based on the PRISMA (Preferred Reporting Items for Systematic Reviews and Meta-Analyses) system (see Checklist 1 for the PRISMA checklist) [24]. The search was conducted on January 2024 by a researcher (DIJ), accessing app stores via mobile devices (Apple iPhone SE and Google Pixel 4a). The review used a series of keyword combinations (n=116) through the Apple App store and Google Play store. The search terms included “pediatric,” “kid,” “teen,” “youth,” “adolescent,” “child,” “infant,” “little,” “minor,” “onco-,” “teenager,” “young,” “blood,” “bone,” “leukemia,” “lymphoma,” “oncology,” “tumor,” and “cancer.”

### Inclusion Criteria

The coauthors (MSSF, MAS, ES, and DIJ) reviewed apps for eligibility. For inclusion in the review, apps met the criteria, which are (1) specifically related to supporting pediatric patients with cancer or families; (2) available in the Apple App store or Google Play store (Android) as of January 30, 2024; and (3) available in English (not exclusively). Free apps (no cost), apps for a fee and free trial apps (freemium), and subscription service apps were included. Apps that functioned as multi-institutional patient portals were excluded for being too broad to be considered a service for pediatric oncology care. A total of 196 apps were identified in both app stores. At the screening round, 159 apps were excluded due to not meeting our inclusion criteria based on store descriptions. Then, the eligible apps (n=37) were assessed for full inclusion (downloading and reviewing the apps). At this stage, we excluded 15 additional apps as they were identified as not being specific to the target population (n=7), not having an English interface (n=1), or were no longer available in either app store by the time the researchers initiated the analysis (n=7).

### Data Collection and Analysis

Data were collected on the variables of descriptions, app rating (out of 5), number of reviews, total cost of services, intended user demographic, file size, supported languages, app privacy and data access requests, latest updates, and the app features from the app stores. App features were determined by reading the app description, reviewing snapshot images provided in the store, downloading the apps from each store on one of the mobile devices (Apple iPhone SE or Google Pixel 4a), and reviewing the original publisher’s website post or press release. Each app was categorized by the level of access required before using the app (no account required, log in via account, closed sign-ups, and shutdown). A secure set of credentials was used for “login via account” apps. Data were recorded on a Microsoft Excel sheet for analysis (see Multimedia Appendix 1).

### App Quality Assessment

We used the Mobile Application Rating Scale (MARS), which is a 23-question assessment of mHealth interventions, to measure apps in 5 domains including engagement, functionality, aesthetics, and information (see Textbox 1) [25]. We used information from the store page and in-app information to evaluate the population of eligible apps. We included 17 apps for MARS evaluation. Apps unavailable for download or that had special access requirements (not publicly available; n=5) were not evaluated. To measure credibility (the legitimacy of the app publisher) and evidence (scientific reports on the test or trial of the app) subcategories in the MARS, apps were cross-verified with external sources from the store page and in-app info, such as the developer’s company page or ClinicalTrials.gov (as suggested by MARS guidelines). A total of 2 researchers evaluated the apps using the MARS instrument and resolved any disagreements via discussion and consensus. Finally, we reported the descriptive results. All included apps were available in the Apple App store but not in the Google Play store (n=5). For those available in both stores, we observed no difference in the user interface; therefore, apps were downloaded and tested on an iPhone SE for the quality assessment. Please see Multimedia Appendix 2 for the MARS scoring for each app.

Textbox 1. **Mobile Application Rating Scale (MARS) domains.**

- Engagement: This domain measures how appealing, flexible, and well-targeted an apps for the target audience. This may include techniques for entertainment (ie, gamification), app features (ie, sound, content, notifications), and applicability between caregiver and pediatric patient age groups.
- Functionality: This measures basic app functions, ease of use, navigational difficulty, and gestural components. This may include how quickly buttons and menus react to user inputs. Additionally, this measures how logically consistent device-specific interactions are, such as taps, swipes.
- Aesthetics: This assesses the layout, graphical design, and visual appeal of the app. This may include quality of graphics or size of the visuals.
- Information: This domain evaluates the quality, quantity, and credibility of information within the app. This includes understanding the source of the content such as the developer and sponsor from their linked store page as well as scientific literature.

## Results

### Overview

**Figure 1.**
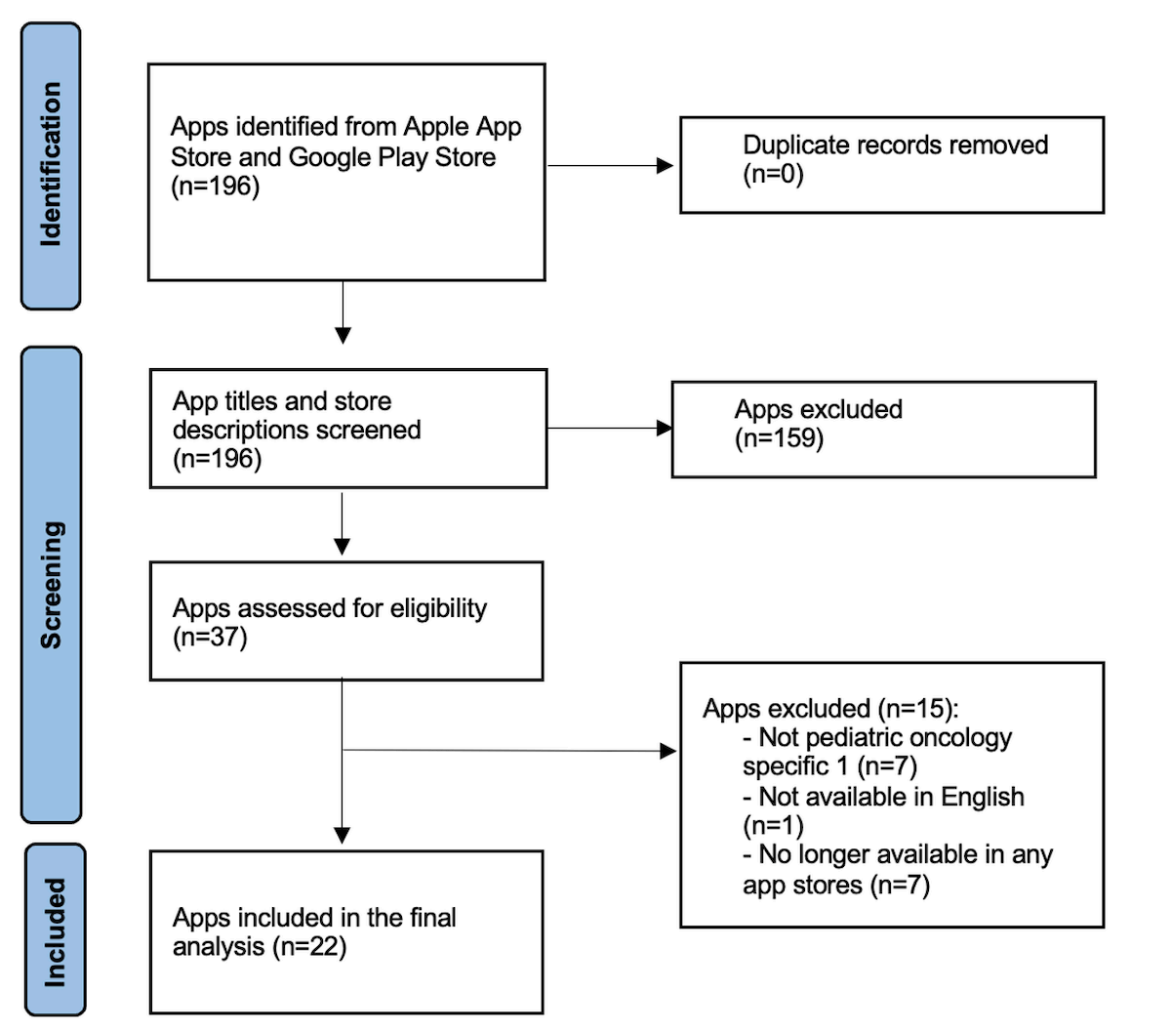
PRISMA flow diagram. PRISMA: Preferred Reporting Items for Systematic Reviews and Meta-Analyses.

In total, 22 apps were included in this review of pediatric cancer apps within the digital marketplace. Figure 1 illustrates the PRISMA flow diagram of the review. Most apps were available in both the Google Play (Android) and Apple App stores and shared the same features for both Android and iOS users (see Multimedia Appendix 1). Therefore, our report is based on consolidated data (Google Play or Android and Apple App store) for each app. More specifically out of 22 apps, 17 of them were available on both marketplaces. The remaining 5 apps were exclusively present on the Apple App store either because the Google Play store (Android) version was taken down or was never created. No apps were exclusively available on the Google Play store.

### App Features

We grouped and summarized the features of the apps by frequency (see Table 1). The resource and information feature was the most frequent property (n=17, 77%). This feature allows users to access guidebooks and video course material related to pediatric cancer. Following that, the symptom tracking feature (n=12, 55%) allowed users to log journal entries or short reports on their immediate symptoms. Reminders (n=10, 45%) let users customize push notifications for important treatment-related events. Connections to care teams, journaling, and community support (n=9, 41%) provided speed-dial options for oncological health care, a logging feature for clinical guidance, and a social component to connect with other pediatric patients with cancer or caregivers directly. Medication tracking and data visualization or graphs (n=8, 36%) saved or described dosage instructions and created pictures to describe variables over time such as medication adherence. The ability to share data, appointment tracking, and health activity data (n=6, 27%) presents a repository for users to save important documents to send pictures to a health care team; schedule upcoming medical appointments on the app calendar; and record basic aerobics, mindfulness activities, and nutritional habits. Integration with other health apps (n=2, 9%) allowed the platform to access other downloaded health and fitness data tracking apps in the patient or caregiver’s phone, such as Apple Health or the Fitbit App (Google).

Most apps (n=16) were free to use and publicly available. “Tracker, Reminder - CareClinic” had additional in-app purchases (up to US $60 total) that expanded on the existing features of the software.

**Table 1.** Apps and feature distribution.

App names	App features												
	Symptom tracking	Medication tracking	Appointment tracking	Information or resources	Push notification reminders	Data visualization or graphs	Ability to share data	Journaling	Connection to care team	Health activity data tracking	Integration with other health apps	Community support	Multiple language support
COG KidsCare	**✓**	**✓**	**✓**	**✓**	**✓**		**✓**	**✓**	**✓**				**✓**
Cancer.Net Mobile	**✓**	**✓**		**✓**		**✓**			**✓**				
My Cancer Tracker	**✓**	**✓**	**✓**			**✓**	**✓**	**✓**					
CancerAid	**✓**	**✓**	**✓**	**✓**				**✓**	**✓**			**✓**	
NET Cancer Health Storylines	**✓**	**✓**		**✓**	**✓**	**✓**		**✓**		**✓**	**✓**		
Pain Squad^a^	**✓**				**✓**								
iThrive Beyond Peds Cancer^a^	**✓**			**✓**	**✓**								
HomeTown Cancer Predisposition	**✓**		**✓**	**✓**	**✓**	**✓**				**✓**			
Heroes Circle^a^												**✓**	
The Breath Brake App^a^												**✓**	
Kids’ Guide to Cancer				**✓**								**✓**	**✓**
The Lounge at MSK				**✓**			**✓**		**✓**			**✓**	**✓**
BELONG beating Cancer Together				**✓**	**✓**		**✓**		**✓**			**✓**	**✓**
Tracker, Reminder - CareClinic	**✓**	**✓**	**✓**		**✓**	**✓**	**✓**	**✓**	**✓**	**✓**	**✓**		
Outcomes4Me Cancer Care	**✓**	**✓**	**✓**	**✓**	**✓**	**✓**			**✓**			**✓**	
iaya				**✓**				**✓**				**✓**	
I’ll explain it to you^a^				**✓**									**✓**
FORTEe Get Strong				**✓**						**✓**			
AYABytes	**✓**			**✓**	**✓**	**✓**		**✓**		**✓**			
Our Journey with Cancer				**✓**				**✓**	**✓**				
LLS Coloring with Kids				**✓**									
OncoPower	**✓**	**✓**		**✓**	**✓**	**✓**	**✓**	**✓**	**✓**	**✓**		**✓**	
Frequency, n (%)	12 (55)	8 (36)	6 (27)	17 (77)	10 (45)	8 (36)	6 (27)	9 (41)	9 (41)	6 (27)	2 (9)	9 (41)	5 (23)

a Only available in Apple App store.

### App Quality Assessment

For each app, we calculated the mean and SD values of MARS scores under 4 categories (Table 2). In addition, we report “objective score,” which represents the mean value of 4 categories. Functionality (mean 3.66, SD 1.05) scored the highest among the 17 apps following aesthetics (mean 3.51, SD 1.02), information (mean 3.49, SD 0.80), and engagement (mean 3.02, SD 1.05). See Multimedia Appendix 2 for detailed scoring in each category.

**Table 2.** App evaluations with the Mobile Application Rating Scale^a^.

App names	Engagement, mean (SD)	Functionality, mean (SD)	Aesthetics, mean (SD)	Information, mean (SD)	Objective score, mean (SD)
COG KidsCare	3.80 (0.98)	4.50 (0.50)	4.33 (0.47)	4.67 (0.75)	4.33(0.82)
My Cancer Tracker	3.00 (1.10)	4.25 (0.43)	4.00 (0.82)	3.17 (0.69)	3.50 (0.96)
CancerAid	2.60 (1.36)	4.25 (0.83)	3.67 (0.94)	4.17 (1.07)	3.67 (1.29)
Net Cancer Health Storylines	4.00 (1.10)	4.50(0.87)	4.00 (0.00)	4.20 (0.75)	4.18 (0.86)
Pain Squad	4.00 (0.63)	4.75 (0.43)	4.00 (0.82)	4.14 (0.99)	4.21 (0.83)
HomeTown Cancer Predisposition	1.60 (0.80)	4.00 (0.71)	3.00 (0.82)	4.00 (1.22)	3.06 (1.39)
Heroes Circle	1.80 (0.75)	1.50 (0.50)	2.33 (0.47)	2.25 (0.43)	1.94 (0.66)
The Breath Brake App	1.80 (0.75)	1.75 (0.83)	1.67 (0.47)	1.60 (0.80)	1.71 (0.75)
Kid’s Guide to Cancer	2.80 (1.33)	3.75 (1.30)	5.00 (0.00)	4.00 (0.89)	3.76 (1.31)
BELONG Beating Cancer Together	4.40 (0.80)	5.00 (0.00)	4.33 (0.94)	3.40 (1.02)	4.24 (1.00)
Tracker, Reminder – CareClinic	4.60 (0.49)	2.50 (0.50)	4.67 (0.47)	3.00 (1.41)	3.65 (1.28)
Outcomes4ME Cancer Care	3.00 (0.89)	4.75 (0.43)	4.00 (0.82)	4.33 (1.11)	4.00 (1.11)
I’ll explain it to you	1.80 (0.98)	4.75 (0.43)	2.33 (0.47)	4.00 (1.26)	3.24 (1.52)
FORTEe Get Strong	3.20 (1.17)	2.25 (0.43)	2.67 (0.47)	2.67 (0.75)	2.72 (0.87)
Our Journey with Cancer	1.40 (0.80)	3.00 (0.00)	2.00 (0.00)	3.40 (0.49)	2.47 (0.98)
LLS Coloring for Kids	4.60 (0.49)	4.25 (0.43)	3.67 (0.94)	3.67 (0.75)	4.06 (0.78)
OncoPower	3.00 (1.41)	3.50 (0.50)	3.00 (0.82)	2.80 (1.60)	3.06 (1.26)
Average score	3.02	3.72	3.45	3.50	3.40

a Objective score reflects average of 1 through 4 for each app.

### Audience Categories

Apps were split into 3 groups based on their intended audience. In the first group, apps that focused on a pediatric population, between the ages of 0 and 17 years (n=7), used activities designed to interest the younger demographic to convey valuable information or make certain features more accessible from a medical literacy perspective. In the second group, apps that focused on caregivers, 18 years and older (n=9), were more likely to create direct connections to health care and generate data visualizations to translate numbers into meaningful interpretations. In the last group, the rest of the apps targeted both pediatric patients and caregivers (n=6). These apps proposed separate user accounts to differentiate parent and child, as well as the targeted features.

### User Ratings and Reviews

The majority of apps (n=18) had user ratings publicly available for review and analysis. A total of 4 apps did not receive any user reviews or ratings (Hometown Cancer Predisposition, Kid’s Cancer Guide, AYABytes, and FORTEe Get Strong). These apps had between 1 and 100 posted reviews per app, whereas there were apps that received a high number of user ratings, such as “Tracker, Reminder - CareClinic” (n=3100) and “BELONG Beating Cancer Together” (n=5770). A total of 16 apps received high ratings (mean 4.4, SD 0.59; Min=3.1, Max=5.0).

### Downloads and Storage

CancerAid, Cancer.Net Mobile, BELONG Beating Cancer Together, and My Cancer Tracker were downloaded by more than 5000 users. The rest of the apps were downloaded by fewer than 500 users. Apps required between 1.7 and 235.3 MB of storage for saving health-related information and app functionality (mean 67.03 MB, SD 58.38 MB).

### Language Availability

To be included, apps were required to have an English option. The majority of the apps provided English exclusively (n=12). However, 10 apps provided multilanguage options including Spanish (n=4), French (n=4), Arabic (n=2), German (n=2), Chinese (n=2), Hebrew (n=1), Dutch (n=1), Italian (n=2), Hindi (n=1), Romanian (n=1), and Portuguese (n=1).

### Data and Health Information Privacy

Apps typically request access to key hardware or software features built into the device to exercise the full length of capabilities designed into the mHealth app. These requests appear as pop-up notifications that require input before continuing to use the app (mean 2.72, SD 3.13; Min=0, Max=10). The types of requests across both app stores included approved access to the calendar, files, camera, microphone, location, user ID, device ID, Wi-Fi networks, contacts, and phone status. Based on our observation, none of the apps mentioned any type of encryption on the app store or in-app. Similarly, none of the apps provided 2-step verification options or similar security measures after account creation.

## Discussion

### Principal Findings

The principal findings of our systematic search and analysis study reveal a noticeable scarcity of pediatric oncology-specific mHealth apps in the digital marketplace, highlighting a critical gap in resources aimed at supporting pediatric patients with cancer and their families. Despite the growing prevalence of mHealth solutions in the broader health care landscape [26], our analysis underscores the underrepresentation of pediatric patients with cancer in this technological advancement [27]. Similarly, Jupp et al’s [28] earlier review identified only 1 out of 28 qualifying oncology apps that specifically served pediatric patients.

The existing apps predominantly focus on educational resources, symptom tracking, and medication reminders. We observed that the adoption of these apps may rely on how well an app can both target specific diagnoses and remain applicable to the wider oncology audience. Therefore, high functionality may have come at the cost of aesthetics and engagement quality of the apps and lacking concise and quality information. While these features align with general needs within chronic illness management, they often lack the specificity and depth required for the complex care trajectories typical in pediatric oncology. This includes focusing on cancers more common in children than adults, implementing risk-based medical follow-ups, a systematic plan for lifelong surveillance, managing symptoms, addressing developmental delays or educational disruption, and mitigating long-term effects of treatment [29,30].

### Quality Assessment

Of the objective domains, functionality scored the highest while engagement scored lowest on average. The prioritization of equipping patients and their caregivers with accessible tools is a strong theme among newer mHealth apps, especially in pediatric oncology. These health management apps can create the structure needed for caregivers and patients to monitor progress, which leads to better accountability and overall better patient outcomes [31]. However, the design of these apps often does not incorporate tactics to maintain attention or consistent usage, such as gamification, in the long term. This in combination with the lower information scores creates an obstacle to sustained adoption. An app may be highly functional and aesthetically professional but lacks specific flexibility and informative quality for caregivers, patients, or both.

Lower scores for engagement are consistent with other studies in the literature that used MARS for oncology app interventions [32]. Additionally, the developers’ tendency to focus on implementation rather than building evidence-based features highlights a trend of understudied interventions on the market today [33]. Many of the apps included in this study either were minimally tested in a usability trial or were not rigorously tested at all. New users of these apps may find oncology mHealth apps helpful for minor tracking purposes like notifications but might find them problematic with evolving treatment plans, expanding diagnostic information, and available support groups in the area.

### Target Population

Of the few apps currently available for pediatric cancer, apps were designed to target children and adolescent patients, caregivers, or both user populations. Apps designed for adolescents and young adults (AYA) with cancer are of interest since nearly two-thirds of AYAs within the United States report using an app for health behaviors, including medication reminders [34]. Several studies have reported the informational needs of AYAs in a cancer care app by highlighting features such as free-text diaries. The overarching goal of a cancer care app is expected to help monitor the impact of the disease and treatment in their day-to-day life and emotions [14]. Additionally, there is a need for personalized data to be adapted to a patient’s specific condition, considering factors such as the type, history, and severity of cancer [27], as well as age-appropriate content that addresses topics like diagnosis, treatment options, sustaining social ties, and strategies to manage the illness [35]. These abilities help AYAs to be more independent with self-care, thus easing the transition to adulthood and long-term survivorship [36]. Additionally, more recent pediatric oncology literature has called out the gap in the child’s voice, particularly in symptom assessment [37-39]. mHealth apps designed specifically for children could provide that opportunity to improve reporting standards.

### Value of Apps in Patient Care

In our study, the majority of the apps focused on educational (information) resources for different user audiences. This aligns with Vaffis et al’s [30] finding as mHealth apps focus on cancer as an important component of patient disease management. Moreover, this is an expected finding as pediatric cancer treatment requires complex treatment regimens, daily medications, intensive side effects, and symptom burden [40]. In addition, medication tracking, symptom tracking, and notifications for reminders are other major features. This matched with the need and also the major challenge to the oncological treatment plans, which is managing medication nonadherence or noncompliance. Missed treatments during home care are major causes of increased adverse outcomes including infection, relapse, and death in this vulnerable population [41]. Therefore, the apps have been aiming to address this vital issue via those critical features, aligning with earlier apps [13]. In addition, the literature presents evidence to support the efficacy of digital interventions in improving medication adherence, psychosocial well-being, and health outcomes in children and adolescents with chronic health conditions [5]. This indicates that mHealth apps aim to improve adherence, self-management and alleviating symptom burden could be essential to improving the use of the apps, and health outcomes.

### Inclusivity

Furthermore, our findings highlight a significant language barrier, with more than half of the reviewed apps available exclusively in English. The accessibility of these digital interventions in alternative languages is a step toward closing the gap in care [42,43]. However, this has been an improvement as a review in 2017 cited that only 20% of all medication apps were offered alternative languages [36]. In addition, the digital divide between socioeconomic and ethnic groups reduces the availability of such resources to underserved populations including limited English proficiency patients and families [44,45]. New approaches via mobile apps should be considered, as these platforms can help with medical literacy and build self-care routines among patients and families [46,47]. In addition, developers and decision makers should consider device compatibility and dependency on cellular connectivity to reduce problems due to inconsistent service, limited storage, data plan requirements, or budget-friendly smartphones that are outside of the regularly maintained cycle of software updates [14,20,36]. Such an approach may support scalable, accessible, and affordable use.

### Privacy and Security

Finally, data security and privacy are important as patients and families are storing and sharing personally identifiable information and confidential health information via apps. Health care institutions create guidelines for handling sensitive data; however, the privacy and security of personal health apps remain the responsibility of end users. Unfortunately, the Health Insurance Portability and Accountability Act (HIPAA) does not regulate third party apps or their services [48]. Other regulations such as the Children’s Online Privacy Protection Act (COPPA) have been a major piece of legislation for protecting child information from third-party organizations, yet it has limited protections with regard to health care information [49-51]. To ensure adequate protection, app stores hosting mHealth tools should have additional protocols to require justification for the necessity of accessing requested phone sensors and other sensitive health informatics (eg, camera, location, and microphone) [48].

### Limitations

This review provided an overview of pediatric cancer-specific apps limited to the currently available apps in the Google Play and Apple App Store. We included these 2 common app stores because they are available in 2 major smartphone operating systems (iOS and Android) and are accessible by the majority of end users [52]. We have not included other stores, such as the Galaxy Store or Amazon Appstore, due to their limited user base and specific requirements for service (ie, the Amazon Appstore requires additional app installations) which may not be applicable for a broader audience of pediatric patients with cancer and caregivers. We focused on the US market for available apps as both app stores are regionally locked due to our physical location. In addition, we focused on apps available in the English language with additional language options. That limited our access to other apps that do not include English language as an option, or alternative apps for different regions or languages. We were not able to use content or sentiment analysis with all user comments because not all apps had a sufficient volume of comments to be analyzed. In addition, we have not received feedback from developers, patients, and clinicians about the apps during the study. This may have limited the study’s objectivity by not including their insights about the apps. Finally, the apps have a life cycle and are subject to change depending on developer updates and business or are not accessible and have restricted access. Some apps could be also removed from these stores for any reason, which may reduce the ability to evaluate these apps continuously and replicate this review with the same set of apps.

### Future Works

Further research is suggested to investigate how developers create mHealth interventions through theory-based frameworks and collaborations (ie, co-design). Based on the MARS, we recommend future interventions to balance focus between extensive customizability and reliable intractability.

Additionally, future development efforts must prioritize the involvement of pediatric oncology stakeholders, including patients, families, and health care professionals, to ensure that the apps are not only technically sound but also deeply aligned with the specific needs, reducing linguistic and cultural barriers. We suggest a focus on evidence-based implementations and rigorous testing approaches for intervention, validation, adoption, and effectiveness. Going beyond, future research may expand how these apps are created from the developer’s perspective and ways to enable cross-disciplinary collaborations including patient and clinician stakeholders as well. Other avenues of research may also incorporate recent innovations in virtual reality, extended reality, and artificial intelligence to create more effective mobile and web apps.

### Conclusions

Our study examined the landscape of mHealth apps for pediatric cancer. While mHealth apps hold promise for enhancing care and support for pediatric cancer treatment, our findings underscore the need for more inclusive, comprehensive, and integrated digital health solutions. The complexity of pediatric cancer is a multifaceted challenge, and mHealth apps can bridge the gaps to become a fundamental source of support for patients and caregivers from diagnosis to survivorship.

## Supplementary material

10.2196/58101 Multimedia Appendix 1 mHealth app list.

10.2196/58101 Multimedia Appendix 2 Mobile Application Rating Scale scores.

10.2196/58101 Checklist 1 PRISMA checklist.

## Abbreviations

AYA: adolescents and young adult
COPPA: Children’s Online Privacy Protection Act
HIPAA: Health Insurance Portability and Accountability Act
MARS: Mobile Application Rating Scale
mHealth: mobile health
PRISMA: Preferred Reporting Items for Systematic Reviews and Meta-Analyses
